# Association between Sexual Habits and Sexually Transmitted Infections at a Specialised Centre in Granada (Spain)

**DOI:** 10.3390/ijerph17186881

**Published:** 2020-09-21

**Authors:** Raquel Casado Santa-Bárbara, César Hueso-Montoro, Adelina Martín-Salvador, María Adelaida Álvarez-Serrano, María Gázquez-López, María Ángeles Pérez-Morente

**Affiliations:** 1 Gregorio Marañón Hospital, 28007 Madrid, Spain; raquelcasado60@hotmail.com; 2 Department of Nursing, Faculty of Health Sciences, University of Granada, 18016 Granada, Spain; 3 Department of Nursing, Faculty of Health Sciences, University of Granada, 52005 Melilla, Spain; 4 Department of Nursing, Faculty of Health Sciences, University of Granada, 51001 Ceuta, Spain; adealvarez@ugr.es (M.A.Á.-S.); mgazquez@ugr.es (M.G.-L.); 5 Department of Nursing, Faculty of Health Sciences, University of Jaén, 23071 Jaén, Spain; mmorente@ujaen.es

**Keywords:** sexually transmitted infections, sexual behaviour, sexual health

## Abstract

Sexually transmitted infections are an important public health issue. The purpose of this study is to analyse the association between different sexual habits and the prevalence of sexually transmitted infections in the population of Granada who consult with a specialised centre. An observational, cross-sectional study was conducted based on the medical records of 678 people from the Sexually Transmitted Diseases and Sexual Orientation Centre of Granada, who were diagnosed positively or negatively with a sexually transmitted infection, during the 2000−2014 period. Sociodemographic and clinical data, as well as data on frequency and type of sexual habits, frequency of condom use and sexually transmitted infection positive or negative diagnosis were collected. Univariate and bivariate analyses were conducted. The most popular sexual habits were vaginal intercourse, oral sex (mouth–vagina and mouth–penis) and the least popular were anus–mouth and anal sex. The use of condom is frequent in vaginal and anal sex and less frequent in oral sex. Sexually transmitted infection is associated with mouth–penis (*p* = 0.004) and mouth–vagina (*p* = 0.023) oral sex and anal sex (*p* = 0.031). It is observed that there is a relationship between the presence of STIs and oral sex practices, people having such practices being the ones who use condoms less frequently. There is also a relationship between anal sex and the prevalence of STIs, although in such sexual practice the use of condom does prevail.

## 1. Introduction

Risky sexual habits and behaviour strongly affect public health. According to the WHO, more than one million people acquire a sexually transmitted infection (STI) every day. The most recent averaged data indicate that one out of 25 people has at least one STI, maybe being infected with several of them at the same time [1].

Complications derived from an STI have a great impact on children’s, teenagers’, and adults’ health and life on a worldwide basis, thus compromising their quality of life. Furthermore, they facilitate indirectly the transmission of HIV and cause cellular changes that lead to some types of cancer. They also impact on domestic economy and national health systems [2].

STIs are more common in people having high-risk sexual behaviour and attitude such as having sexual intercourse without using condoms, having contact without buccal–genital protection, having multiple sex partners, having a high-risk partner (someone having many sex partners or other risk factors), having anal sex or a partner who has an STI, having sexual intercourse with sex workers, or having sexual intercourse with someone who injects or has injected drugs before [3,4]. Moreover, an increase in the use of the Internet and mobile applications to easily meet sexual partners, thus increasing high-risk sexual practices, should be highlighted [5,6].

Hence, it is important to know the population’s sexual habits in order to prioritise sexual health strategies that shall reduce the incidence of STIs, and therefore reduce the morbidity and mortality load worldwide derived from sexually transmitted pathogens.

Research has focused on studying the use of condom and its effectiveness in people with high-risk sexual contact [7,8,9], however, there are only a few studies on general population, which show that they are less aware of risks and motivation for condom use decreases [10,11]. In the United States, among 15−44-year-old people, prevalence of anal intercourse slightly increased from 2002 to 2015 both in women (30–33%) and men (34–38%), condoms being used in 32% in women and 46% in men, and only 7% during oral sex; such low percentages regarding condom use in high-risk sexual practices lead to an increase in the likelihood of getting an STI [12].

In Spain, a Blanc et al.’s study [13] revealed that the use of condom is higher in vaginal intercourse, and diminishes in anal sex and it is used in a lower percentage during oral sex (29.2%, 17.0%, and 2.0%, respectively). These results support the idea that young people use condoms more to avoid pregnancy than to prevent STIs.

Most published studies address sexual habits, condom use, and prevalence of STIs independently [14,15,16]; therefore, the purpose of this study is to analyse the association between different sexual practices and an STI diagnosis in people consulting with the Sexually Transmitted Diseases and Sexual Orientation Centre of Granada (Spain).

## 2. Materials and Methods

An observational, retrospective and cross-sectional study was conducted based on the medical records of people consulting with the Sexually Transmitted Diseases and Sexual Orientation Centre of Granada, attached to the Andalusian Health Service (Spain). It is the STI reference centre in the Granada province, it belongs to the public health system and it carries out preventive, diagnoses-related and therapeutic activities.

The sample was taken from a database composed of 1437 medical records of subjects attending for consultation associated with the presence or suspicion of an STI between 2000–2014. The sampling and data collection process may be checked in detail in a previous publication [17]. From said database, 678 medical records were taken for this research, which records complied with the criterion of having a test made to confirm or rule out the presence of an STI. Each history corresponds to one subject, so that there is no duplicate information in the final case selection. Every history was reviewed by a member of the research team, who took out data to include them in a data collection sheet created to that effect for this study. One of the disadvantages of this process was related to missing data, so the sample sizes defined for every analysis performed are specified in the data results.

The STI diagnosis was considered a dependent variable, collected as a binary qualitative variable (Yes, No). Sexual habits were established as an independent variable, identifying five practices: vaginal intercourse, oral (mouth–vagina), oral (mouth–penile), oral (anus–mouth), anal (penile–anus) intercourse. Subjects were asked about the frequency of such practices, so every habit was collected as a binary qualitative variable (Never or sporadic, Frequent or always). Additionally, data on condom use were collected for each and every described practice, following the same categorization in answers. To classify the sample, sociodemographic variables (age, sex, nationality, occupation, working status, education level, marital status, and sexual orientation), medical variables (reason for consultation) and risk indicators (age of first sexual intercourse, last time having sex without a condom, number of partners in the last month and in the past 12 months, use of drugs, and prior STI) were collected.

A univariate analysis was conducted by calculating the median (Me), interquartile range (IQR), frequency (*n*), and percentage (%), according to the variable type. The bivariate analysis was performed in order to analyse the association between STIs (dependent variable) and sexual habits (independent variable), via the chi-square test, by calculating the effect size through the Cramer’s V (V), plus the Odds ration (OR) (Confidence Interval (CI) 95%).

In every case, a significant association was considered with *p* < 0.05. Univariate and bivariate analyses were conducted using the Statistical Package for the Social Sciences (SPSS) program, version 22, (IBM, New York, NY, USA).

The study protocol was approved by the Biomedical Research Ethics Committee of the province of Granada (research protocol approved on 12 February 2012, and 1 April 2015), as well as by the Management Directorate of the Granada-Metropolitan Health District, to which the centre where data were collected is attached.

## 3. Results

Table 1 shows results corresponding to sociodemographic variables, medical care, and risk indicators. The general profile of the sample was a young subject, Spanish, with higher-level education, employed, mostly single, and heterosexual. The sample included a similar proportion of men and women, although with a slightly higher percentage of men. They consulted with the STI centre for some reason related to STI contagion or suspicion, highlighting the presence of symptoms as the reason for consultation.

Out of 678 analysed cases, 65.5% (*n* = 444) got an STI positive diagnosis, as opposed to 34.5% (*n* = 234) of negative cases. Table 2 shows the results of sexual habits and condom use variables in such sexual practices.

Table 3 shows the results corresponding to the association between STIs and sexual habits. STI diagnosis was significantly associated with the practice (frequent-always) of mouth–penile oral sex (*p* = 0.004), mouth–vagina oral sex (*p* = 0.023), and anal sex (*p* = 0.031). In the three cases, the effect size was low.

## 4. Discussion

From among the results of this research, it is worth mentioning the association observed between the STI diagnosis and the frequency of oral sex (mouth–penile and mouth–vagina) and anal sex. Before describing such finding more deeply, there are aspects of the sample features that should be pointed out.

The age of the first sexual intercourse was around 17 years old, which approximates to other studies which establish the commencement of sexual intercourse between 17 and 18 years old [6,18], unlike other studies which point out slightly lower figures, reporting median ages between 15 and 16 years old [19,20]. It has been shown that a reduction in the age of the first sexual intercourse is a factor contributing to an increase in STIs [21,22].

As regards other risk indicators, subjects stated they had one sexual partner or no sexual partner during the last month and approximately 50% stated they had two or fewer partners during the last year, which results are similar to the ones observed in another study [4]. Although our study does not observe an elevated number of sexual partners, an increase in the number of sexual partners is known to be related to an increase in STIs [21,23]. With respect to the use of drugs, most of them stated they did not use or used them sporadically, which findings also coincide with another research [6]. As regards such finding, it should be noted that factors leading to a higher risk of acquiring HIV and STIs include not only early sexual intercourse and a higher number of sexual partners, but also drug use during said intercourse [22,23]. In Europe, an earlier initiation of sexual intercourse is observed, as well as an increase in the number of sexual partners, which would contribute to an increase in the incidence of STIs [21,23].

Regarding the sexual habits analysed, vaginal intercourse stands out as to frequency, followed by mouth–penis and mouth–vagina oral sex. In contrast, there were very few reported cases of mouth–anus oral sex as well as anal sex. Such data are similar to data from another study where higher percentages were found for vaginal intercourse [24], or where oral sex was more frequent than anal sex [18]. Our findings with respect to a higher frequency of vaginal sex as opposed to anal sex may be conditioned by mostly heterosexual subjects in our sample. The importance of anal intercourse as opposed to vaginal intercourse resides in the fact that the former constitutes a higher-risk sexual practice regarding transmission of HIV both for men and women [25].

In relation to condom use, results show an inconsistent use when asking subjects about the time elapsed from the last time they had sex without using a condom, which could increase contagion and transmission of STIs. It is worth mentioning that evidence is firm when relating the low use of condom to a higher risk of getting an STI [13,24,26,27]. In general, the epidemiological studies available has shown that, when condoms are used constantly and correctly, they are highly effective to prevent HIV infection and they reduce the risk of other STIs [28,29].

Upon analysing the use of condom in different sexual practices in detail, it is observed that its use in vaginal intercourse occurs always or frequently, closely followed by its use in anal sex. However, in mouth–vagina and mouth–penis oral sex, the use of condom is non-existent or sporadic. Previous studies consulted have varied results; some of them agree on the use of condom in vaginal intercourse and its non-frequent use in oral sex [13,30], and others report a lower use than the one observed in our study regarding anal sex [13]. Specifically, in the “Encuesta nacional sobre sexualidad y anticoncepción entre los jóvenes españoles” [19] (“National survey on sexuality and contraception among Spanish young people”), the main reason for not always using contraception methods, like condom, resides in the number of occasions in which oral sex is practised (59.1%). A possible explanation for the low use of condom in oral sex could be the fact that people practising it are less aware of the risk of acquiring an STI during oral sex or because they see condoms as a barrier that adversely affects sexual pleasure in the couple.

Our study shows a significant association between mouth–penis and mouth–vagina oral sex and an STI positive diagnosis. As observed, in such sexual practices, the frequency of condom use is low, which could be one of the causes explaining such association. Coincidences with another study were found, which study suggests that many young people are still unaware of the ways in which STIs may be transmitted in oral–genital contact [13] and, although some studies have demonstrated that the risk of getting HIV in oral sex with an infected partner (whether by giving or by receiving it) is much lower than the risk of getting this virus in anal or vaginal intercourse with an infected partner, it is possible that this may not be the case for other STIs [31]; hence, the importance of condom use in oral sex as well.

Furthermore, a significant association between anal sex and an STI positive diagnosis was found; although, it is worth recalling that descriptive data showed this practice is not frequent in the sample studied. Anal intercourse is the riskiest sexual practice as regards transmission of STIs such as HIV, chlamydia, or gonorrhea [32], the risk being higher when a passive role is adopted [33,34], with a probability of infection 13 times higher than for the sexually active partner, due to the fact that the lining of the rectum is thin and may allow HIV to enter the body during anal sex [25].

This study has some limitations, one of them being related to the high percentage of values lost in some variables, since not in all of them data on the same number of subjects were registered; when working with medical records, not all variables are completed. In addition, another limitation is the fact that it is a single-centre and single-province study, which, when extrapolating the results obtained, would limit their external validity. Nevertheless, the WHO has emphasized, in its latest reports, the availability of local data to improve the approach to STIs [2]. Finally, the methodological design used does not allow for the establishment of causal relations, so the associations observed must be considered as hypotheses to be supported by more complex designs and analyses.

## 5. Conclusions

In conclusion, the most frequent sexual practices reported in this research were vaginal and oral (mouth–vagina and mouth–penis) sex, unlike the low frequency of anal sex. Likewise, the use of condom was frequent in vaginal and anal sex, as opposed to the figures observed in oral sex. A statistically significant relationship is established between the presence of STIs and oral and anal sex.

The results obtained in this study may contribute to the design of sex education policies aimed at reducing the risk of certain sexual practices through strategies oriented to an improvement in sexual health and a minimization of exposure to and contagion of STIs. As previously mentioned, the findings of this study are in line with the WHO’s proposal [2] of worldwide strategies to prevent sexually transmitted infections, where disparity in reports among different regions and countries, as well as within every region and every country, is worth mentioning because of the difficulty derived from the lack of local data to globally address STIs, in order to measure the advance towards their control.

## Figures and Tables

**Table 1:** Sociodemographic features, medical care and risk indicators.

Variables	Me (IQR)
**Age (*n* = 678)**	26 (23–33)
**Age at first sexual intercourse (*n* = 322)**	17 (16–18)
	***n* (%)**
**Sex (*n*= 678)**	
Male	391 (57.7)
Female	287 (42.3)
**Citizenship (*n* = 671)**	
Spanish	511 (76.2)
Immigrant	160 (23.8)
**Occupation (*n* = 630)**	
Other occupation	316 (50.2)
Student	229 (36.3)
Sex workers/former workers	85 (13.5)
**Employment (*n* = 615)**	
Active	302 (49.1)
Unemployed	77 (12.5)
Retired	7 (1.1)
Student	229 (37.2)
**Education level (*n* = 640)**	
Without education	4 (0.6)
Primary	116 (18.1)
Secondary	150 (23.4)
Vocational training	70 (10.9)
Higher (University)	300 (46.9)
**Marital status (*n* = 675)**	
Single	544 (80.6)
Married/Domestic partner	92 (13.9)
Separated/Divorced	38 (5.6)
Widower	1 (0.1)
**Sexual orientation (*n* = 657)**	
Heterosexual	547 (83.3)
Homosexual	81 (12.3)
Bisexual	29 (4.4)
Reason for consultation * (*n* = 678)	
HIV	219 (32.3)
STI symptoms	429 (63.3)
Control	20 (2.9)
Follow-up of contacts	10 (1.5)
**Last time having sex without condom (*n* = 385)**	
Never used it	31 (8.1)
Last month	178 (46.2)
1−6 months	147 (38.2)
6–12 months	12 (3.1)
Over 1 year	17 (4.4)
**Partners in the last month (*n* = 636)**	
0−1	465 (73.1)
2	54 (8.5)
3−5	30 (4.7)
+5	9 (1.4)
Sex workers	78 (12.3)
**Partners in the last year (*n* = 633)**	
0−1	229 (36.2)
2	102 (16.1)
3−5	130 (20.5)
6−10	68 (10.7)
11−20	21 (3.3)
+20	10 (1.6)
Sex workers	73 (11.5)
**Drug use (*n* = 288)**	
No	185 (64.2)
Yes	103 (35.8)
**Prior STIs (*n* = 542)**	
No	412 (76.0)
Yes	130 (24.0)

* HIV: Human Immunodeficiency Virus; STI: Sexually Transmitted Infections; Control: people going to the centre for an STI control; Follow-up of contacts: people who go to the centre because they have had a risky contact.

**Table 2:** Sexual habits and condom use.

Variables	Vaginal Intercourse		Oral (Mouth–Vagina)		Oral (Mouth–Penile)		Oral (Anus–Mouth)		Anal (Penile–Anus)	
Frequency	H * *n* (%)	U.C. * *n* (%)	H * *n* (%)	U.C. * n (%)	H * *n* (%)	U.C. * *n* (%)	H * *n* (%)	U.C. * *n* (%)	H * *n* (%)	U.C. * *n* (%)
Never-Sporadic	3 (0.8)	131 (36.8)	43 (22.5)	143 (98.6)	38 (15.3)	193 (86.5)	6 (100)	1 (50.0)	216 (72.7)	61 (43.0)
Frequent-Always	358 (99.2)	225 (63.2)	148 (77.5)	2 (1.4)	210 (84.7)	30 (13.5)	0 (0)	1 (50.0)	81 (27.3)	81 (57.0)
Total	361 (100)	356 (100)	191 (100)	145 (100)	248 (100)	223 (100)	6 (100)	2 (100)	297 (100)	142 (100)

* H = habit; U.C. = use of condom.

**Table 3:** Sexual habits and STI diagnosis.

Variables	STI Yes		STI No		*p*	V	OR (CI 95%)
	*n*	%	*n*	%			
**Vaginal intercourse (*n* = 361)**							
Frequent-always	224	62.6	134	37.4	n.s *	n.a *	n.a *
Never-sporadic	2	66.7	1	33.3			
**Oral (mouth–penile) (*n* = 248)**							
Frequent-always	145	69.0	65	31.0	0.004	0.184	2.756 (1.364−5.567)
Never-sporadic	17	44.7	21	55.3			
**Oral (anus–mouth) (*n* = 6)**							
Frequent-always	0	0.0	0	0.0	n.s *	n.a *	n.a *
Never-sporadic	3	50.0	3	50.0			
**Oral (mouth–vagina) (*n* = 191)**							
Frequent-always	94	63.5	54	36.5	0.023	0.164	2.199 (1.104−4.378)
Never-sporadic	19	44.2	24	55.8			
**Anal (penile–anus) intercourse (*n* = 297)**							
Frequent-always	60	74.1	21	25.9	0.031	0.125	1.854 (1.052−3.268)
Never-sporadic	131	60.6	85	39.4			

* n.s.: not significant; n.a.: not applicable.
